# Galectin 3: association to neurohumoral activity, echocardiographic parameters and renal function in outpatients with heart failure

**DOI:** 10.1186/s12872-016-0290-7

**Published:** 2016-05-31

**Authors:** Freja Stoltze Gaborit, Helle Bosselmann, Caroline Kistorp, Kasper Iversen, Thomas Kumler, Finn Gustafsson, Jens P. Goetze, György Sölétormos, Niels Tønder, Morten Schou

**Affiliations:** Department of Cardiology, Herlev University Hospital, Herlev, Denmark; Department of Internal Medicine KNEA, North Zealand University Hospital, Hillerod, Denmark; Department of Internal Medicine, Endocrinology Unit, Herlev University Hospital, Herlev, Denmark; Department of Cardiology, Rigshospitalet, Copenhagen, Denmark; Department of Clinical Biochemistry, Rigshospitalet, Copenhagen, Denmark; Department of Clinical Biochemistry, North Zealand University Hospital, Hillerod, Denmark; Department of Clinical Medicine, University of Copenhagen, Copenhagen, Denmark

**Keywords:** Heart failure reduced ejection fraction, Galectin 3, Renal function, Echocardiography, Natriuretic peptides

## Abstract

**Background:**

Galectin 3 (Gal-3) reflects cardiac fibrosis in heart failure HF, but has also been associated to renal fibrosis and impaired renal function. Previous research has suggested that Gal-3 could be a cardio-renal biomarker, but it has never been tested simultaneous in a single study whether Gal-3 reflects echocardiographic measures, neurohumoral activity and renal function.

The aim of this study was to evaluate the relationship between plasma concentrations of Gal-3 and neurohumoral activity, myocardial and renal function in patients with HF, including advanced echocardiographic measures and 24-h urinary albumin excretion (albuminuria).

**Methods:**

We prospectively enrolled 132 patients with reduced left ventricular ejection fraction (LVEF) referred to an outpatient HF clinic. The patients had a median age of 70 years (interquartile rage: 64–75), 26.5 % were female, median LVEF was 33 % (27–39 %) and 30 % were in NYHA class III-IV.

**Results:**

Patients with plasma concentrations of Gal-3 above the median had significantly lower estimated glomerular filtration rate (eGFR) and this association remained significant in multivariate regression analysis (β: −0.010; 95 % CI −0.012–-0.008; *P* < 0.001), adjusted for age, gender, medical treatment. Plasma concentrations of Gal-3 were not associated with albuminuria (Beta: 0.008; 95 % CI:-0.028–0.045; *P* = 0.652). There were no association between plasma concentrations of Gal-3 and myocardial function or structure estimated by LVEF, LVmassIndex, LVIDd, E/é or LV global longitudinal strain (*P* > 0.05 for all). In multivariate analyses plasma concentrations of Gal-3 were significantly associated with the cardiac biomarkers: NT-proBNP (β: 0.047; 95 % CI: 0.008–0.086; *P* = 0.020), proANP (β: 0.137; 95 % CI: 0.067–0.207; *P* < 0.001), chromogranin A (β: 0.123; 95 % CI: 0.052–0.194; *P* < 0.001) and Copeptin (β: 0.080; 95 % CI: 0.000–0.160; *P* = 0.049). Multivariate analysis was adjusted for eGFR, age, gender and medical treatment.

**Conclusions:**

Increased plasma concentrations of Gal-3 are associated with reduced eGFR and increased plasma concentrations of NT-proBNP, proANP, chromogranin A and Copeptin, but not with echocardiographic parameters reflecting myocardial function. These results suggest that Gal-3 reflects both increased neurohumoral activity and reduced eGFR, but not myocardial function in patients with systolic HF.

**Electronic supplementary material:**

The online version of this article (doi:10.1186/s12872-016-0290-7) contains supplementary material, which is available to authorized users.

## Background

Galectin 3 (Gal-3) is a soluble β-galactoside-binding lectin. It is expressed in epithelial and inflammatory cells in several organs and it is located both intra- and extracellularly [1, 2]. Gal-3 is involved in cellular functions related to cell adhesion, proliferation and differentiation [3]. Gal-3 has been linked to fibrosis in a spectrum of medical conditions including heart failure (HF) [4–7].

Increased plasma concentrations of plasma Gal-3 are inversely associated to renal function evaluated by estimated glomerular filtration rate (eGFR) in both the general population and in HF patients [8, 9]. Increased plasma concentrations of Gal-3 have predictive value of deteriorating renal function and de novo renal disease [10]. It is, however, unknown whether plasma concentrations of Gal-3 are related to 24 h urine albumin excretion (albuminuria), a renal biomarker which also provides prognostic value in HF [11].

Increased plasma concentrations of Gal-3 are observed in both acute and chronic HF and it has been associated to increased risk of mortality and re-hospitalization [12–14]. Previous studies have suggested an association between plasma concentrations of Gal-3 and NT-proBNP [12, 14]. Plasma concentrations of Gal-3 have also been linked to cardiac fibrosis and it seems reasonable to hypothesize that it would also be associated to echocardiographic measures of left ventricular (LV) function [15, 16].

Thus, the clinical potential of Gal-3 and its pathophysiological aspects are not fully investigated in HF. We, therefore, wanted to evaluate whether plasma concentrations of Gal-3 were associated with I) neurohumoral activity (natriuretic peptides), II) myocardial function estimated by echocardiography and III) renal function evaluated by eGFR and albuminuria.

## Methods

### Population

This study was conducted using data from the CardioRen cohort [17]. Patients were enrolled at their first visit at the outpatient HF clinic at the North Zealand University Hospital, in the period from January 2011 to November 2012. Inclusion criteria were LVEF < 45 %, clinical stable (60 days out of hospital) and stable plasma-creatinine (+/− 10 μM the last 60 days). A total of 149 patients fulfilled all inclusion criteria and agreed to participate in the study. A total of 17 patients were excluded from the analyses due to missing venous blood samples or a missing Gal-3 value. As a result 132 patients with complete data were included in this study. All patients provided written informed consent. The study was approved by the Committee on Health Research Ethics for the Capital Region of Denmark (H-1-2010-074) and was conducted according to the Declaration of Helsinki.

Data were collected at the second visit to the HF clinic, including medical history, present symptoms, functional class, medication, height, weight, non-invasive blood pressure, heart rate, 12-lead electrocardiogram. Additionally venous blood samples were taken and patients collected a 24 h urine sample. Advanced echocardiography was preformed according to standard recommendations of the European Society of Cardiology [18].

### Biomarkers

Hemoglobin and creatinine were analyzed successively for each patient. Plasma samples for later analysis were collected in ethylenediamine tetracetic (EDTA) vial, centrifuged at 4 degrees (3,000 rpm in 10 min) and stored in −80 degrees Celsius.

Plasma concentrations of NT-proBNP, Troponin I and high sensitive c-reactive protein (hsCRP) were measured on the Dimension Vista®1500 from Siemens Medical Solutions Diagnostics using the LOCI®-technology (Luminescent Oxygen Channeling Assay) according to the manufacturer’s instructions [19]. Plasma concentrations of Copeptin were measured on the Kryptor Compact platform (BRAHMS), assay and validation has been reported previously [20]. Plasma concentrations of proANP were measured using an automated immunoluminometric assay (Thermo-Fisher, Hennigsdorf/Berlin, Germany). Plasma concentrations of Chromogranin A were measured with an immuno assay in two steps to quantify the total amount of Chromogranin A in plasma, as previously described [21]. Gal-3 plasma concentrations were measured with a commercial enzyme-linked immunosorbent assay (BG Medicine, Waltham, MA, USA) [22].

### Renal function

Renal function was calculated as eGFR by the Chronic Kidney Disease Epidemiology Collaboration (CKD-EPI) equation incorporating age, race, gender, and plasma creatinine concentration [23]. Impaired renal function was defined as eGFR <60 mL/min per 1.73 m^2^. Patients collected a 24 h urine specimen for albumin excretion analysis (albuminuria, mg/24 h).

### Echocardiography

Echocardiography was performed with the Vivid E9 (General Electric, Vingmed, Horten, Norway) and analyzed offline (Echopac BT 12, 1.0, General Electric, Horten, Norway) by a single trained operator blinded to renal function and plasma concentrations of Gal-3.

LVEF was calculated by the Simpson biplane model. LV mass was calculated from the linear dimensions in the parasternal view and indexed to body surface area according to gender (LVmassIndex). Maximal left atrial volume index was determined from the biplane area method and indexed to body surface area LAESVindex). Mitral inflow was determined by pulse wave Doppler recordings and peak velocity of early (E) and atrial filling (A) and mitral valve deceleration time. Tissue Doppler velocity was measured at both lateral and septal mitral annulus for myocardial peak early velocity (e’) and peak systolic velocity (s’). Mean e’ was calculated from the lateral and septal e’, and used for calculating E/e’. In case of atrial fibrillation mean value of e’ was calculated from three consecutive heart cycles. LV global longitudinal strain (GLS) was assessed by two-dimensional speckle tracking in the three apical projections (long axis, four chambers and two chambers). LV GLS was analysed in 17 standardised segments and total LV GLS was calculated automatically by Echopac software.

### Statistics

Baseline clinical and biochemical data were calculated according to median Gal-3. Dichotomous variables as proportion in percentages and continuous variables as median with interquartile range (25–75 %). The two groups were compared with Pearson Chi square-test for discrete variables and t-tests (parametric) and Mann Whitney U-tests (nonparametric) for continuous variables, as appropriate. Univariate and multivariate linear regressions analyses were performed for the relation between plasma concentrations of Gal-3 and each cardiovascular biomarker and echocardiographic variable, with the cardiovascular biomarker or echocardiographic variable as the response variable (+/−median), in separate models. Explanatory variables (covariates) were chosen from available established confounders: eGFR, age and gender. All other potential confounders (diabetes, NYHA class, LVEF, ischemic heart disease, body mass index, atrial fibrillation) were eliminated by backward elimination (*P* > 0.10) to avoid overfitting of the statistical models. Data quality of covariates included in the statistical models was > 90 %. Results were considered statistically significant when *P* < 0.05 (two-sided).

## Results

Baseline characteristic are presented in Table 1. Patients were divided in two groups with plasma concentrations of Gal-3 above or below median (median Gal-3: 16.90 ng/mL). Impaired renal function was present in 28 % of all patients, defined as eGFR < 60 mL/min/1.73 m^2^. Ischemic heart disease verified by previous coronary angiography was present in 40 %.

**Table 1:** Baseline characteristics according to Galectin 3 plasma concentration above or below median (median = 16.90 ng/mL). Linear variables presented: median (25–75 % percentiles)

Variable	Below median Galectin 3 (*n* = 64)	Above median Galectin 3 (*n* = 68)	*P*-value
Age, years	68 (62–73)	73 (68–78)	0.010
Female sex, %	21.9	30.9	0.241
BMI, kg/m2	26 (24–29)	27 (23–31)	0.191
NYHA class III-IV, %	15.6	44.1	<0.001
Systolic BP, mmHg	130 (113–143)	122 (110–137)	0.225
Diastolic BP, mmHg	77 (70–84)	73 (68–81)	0.174
Heart rate, beats/min	67 (59–75)	68 (60–78)	0.292
HF duration, months	7.5 (6–12)	12 (6–24)	0.257
Medical history:			
Pervious MI, %	46.9	32.4	0.088
Hypertension, %	57.8	64.7	0.405
Atrial fibrillation, %	35.9	32.4	0.664
ICD, %	10.9	13.4	0.663
Diabetes, %	18.8	26.5	0.290
Apoplexia Cerebri/ TCI, %	12.5	22.1	0.148
Medication:			
ACE-I, %	64.1	72.1	0.333
ARB, %	31.7	22.1	0.211
Beta-blocker, %	85.9	85.3	0.916
MRA, %	12.5	26.9	0.039
Blood sample analysis:			
Hemoglobin, mmol/L	8.9 (8.3–9.4)	8.2 (7.6–8.8)	0.004
Creatinine, umol/L	76.0 (66.0–84.0)	98.0 (81.0–130.5)	0.001
eGFR, mL/min/1.73 m2	81.0 (70.0–88.0)	55.0 (40.5–70.0)	<0.001
Galectin 3, ng/mL	13.90 (12.43–14.49)	21.85 (18.65–27.58)	<0.001
NTproBNP, pg/mL	920.15 (393.75–1712.30)	1931.15 (736.20–4059.25)	<0.001
ProANP, pmol/L	726.89 (470.29–1099.33)	1205 (658.80–1627.44)	0.006
Troponin I, % above median	32,8	38,2	0.519
hsCRP, mg/L	1.64 (0.87–3.30)	3.64 (1.61–8.21)	0.001
Chromogranin A, pmol/L	79.0 (49.0–116.0)	106.5 (72.0–240.0)	0.001
Copeptin, pmol/L	7.89 (5.07–12.62)	10.88 (7.03–32.85)	<0.001
Urine Sample analysis:			
Albuminuria, mg/24 h	11.0 (9.0–23.5)	14.0 (8.0–29.5)	0.213

*Abbreviations*: *BMI* Body mass index, *NYHA* New York Heart Association, *BP* Blood pressure, *HF* Heart failure, *MI* Myocardial infarction, *ICD* Implantable cardiac defibrillator, *TCI* Transitory cerebral ischemia, *ACE-I* Angiotensin converting enzyme inhibitor, *ARB* Aldosterone receptor blocker, *MRA* Mineralocarticoide receptor antagonist, *eGFR* Estimated glomerular filtration rate, *NTproBNP* N-terminal-pro Brain Natriuretic Peptide, *proANP* Pro-atrial-natriuretic-peptide, *hsCRP* high sensitive C-reactive protein

**Fig. 1:**
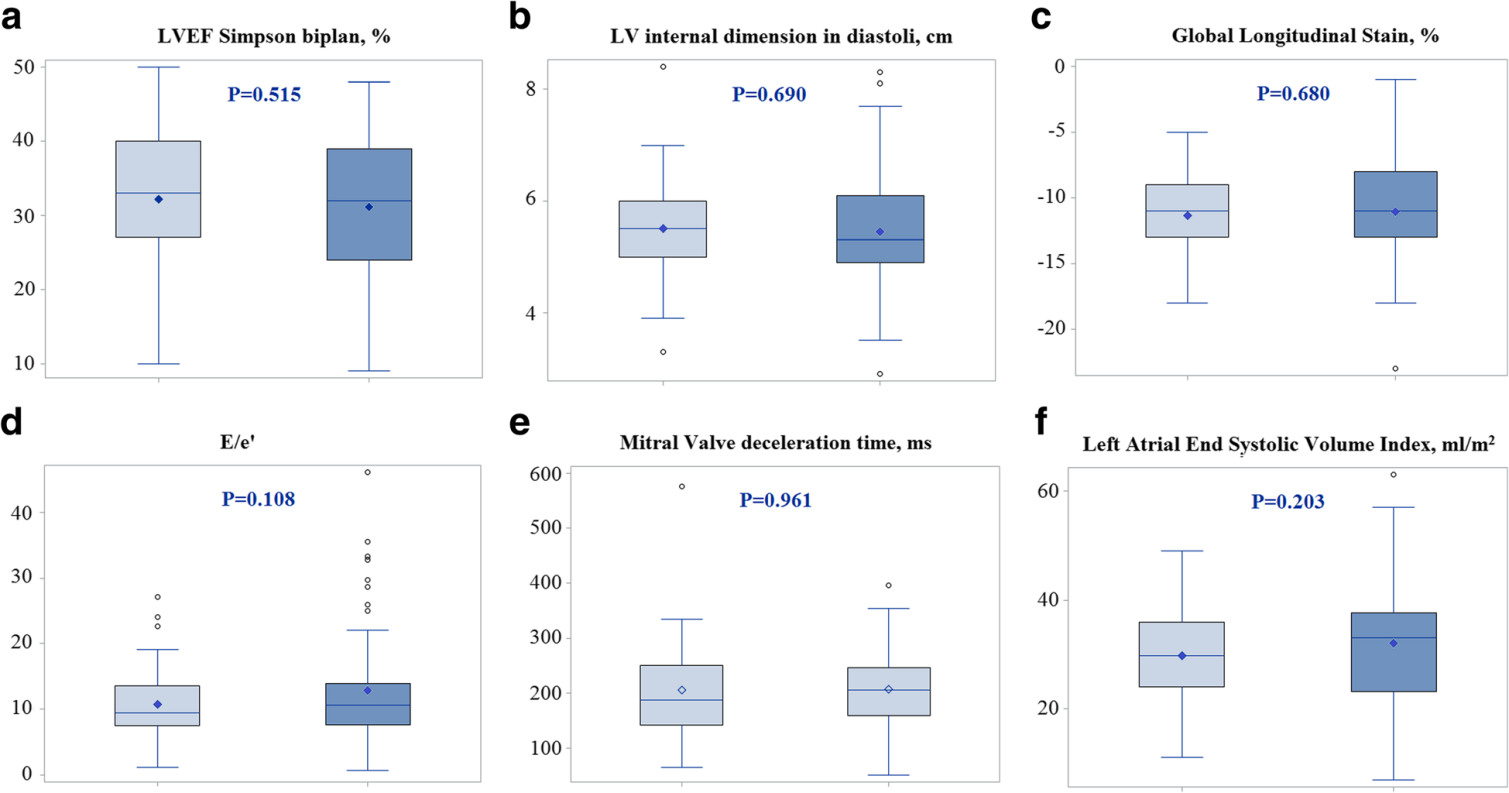
**a**-**f** Box plot of medians of echocardiographic variables according to Galectin 3 plasma concentration above median (*n* = 68) (light blue) or Galectin 3 plasma concentration below median (*n* = 64) (dark blue). Patients with plasma concentrations of Galectin 3 above median had a lower TAPSE and lower s’ medial, other echocardiographic variables did not show signs of an uneven distribution (*P* > 0.05 for all)

Patients with Gal-3 plasma concentrations above the median were older (*P* = 0.010), were more symptomatic (*P* < 0.001) and more often received a mineralocorticoid receptor antagonist (*P* = 0.039) (Additional file 1: Table S1 and Additional file 2). Table 1 also demonstrates that patients with increased Gal-3 plasma concentrations had higher plasma creatinine (*P* = 0.001), lower eGFR (*P* < 0.001), lower plasma hemoglobin (*P* = 0.004) and higher concentrations of NT-proBNP (*P* < 0.001), hsCRP (*P* = 0.001), Copeptin (*P* < 0.001) and Chromogranin A (*P* = 0.001). These findings suggest that patients with Gal-3 plasma concentrations above median suffer from more advanced stages of heart failure. In Fig. 1 echocardiographic variables are presented according to median plasma Gal-3. Patients with Gal-3 plasma concentrations above the median had a decreased TAPSE (*P* = 0.031) and a decreased s’ medial (*P* = 0.022). Other echocardiographic variables did not differ significant between the two groups, this included test for LVmassIndex (*P* = 0.832) and s’ lateral (*P* = 0.473).

**Fig. 2:**
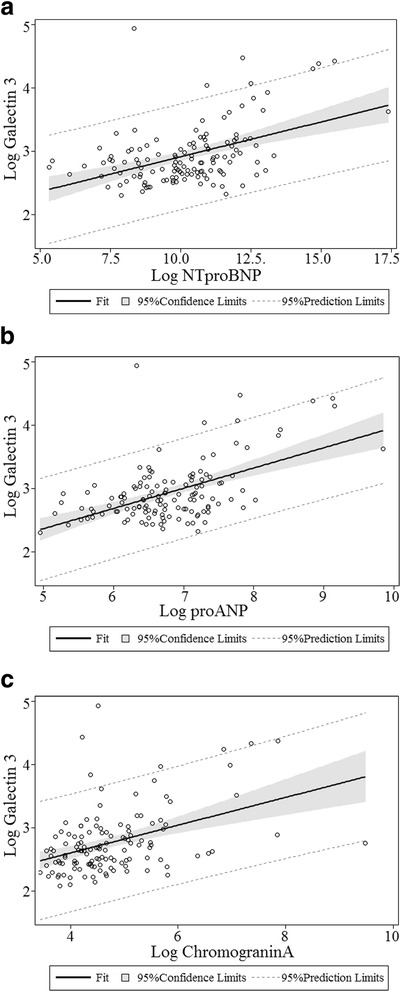
Scatterplot of the correlation between (**a**) Galectin 3 plasma concentration and NT-proBNP; (**b**) Galectin 3 plasma concentration and proANP and (**c**) Galectin-3 plasma concentration and Chromogranin A

**Fig. 3:**
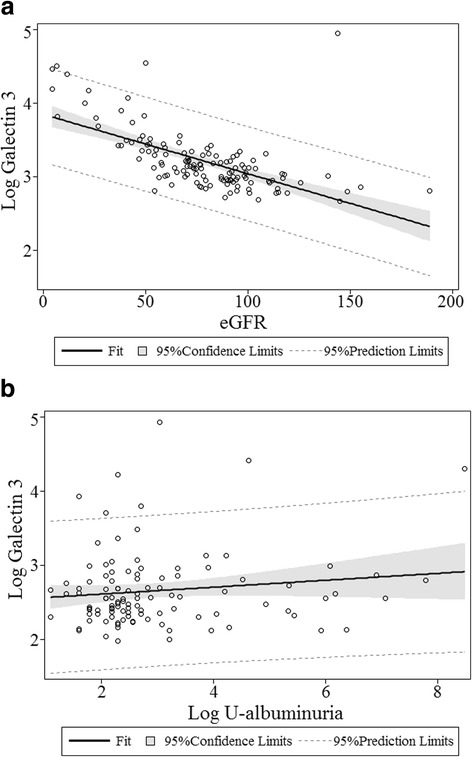
Scatterplot of the correlation between (**a**) Galectin 3 plasma concentration and eGFR and (**b**) Galectin 3 plasma concentration and albuminuria. Plasma concentration of Galectin 3 were significantly associated to eGFR in linear univariate (Beta: −0.009; 95%CI: −0.011–-0.007; *P* < 0.001) and multivariate analysis (β -0.010; 95%CI: −0.012–-0.008; *P* < 0.001), adjusted for age and gender, treatment with beta-blocker, ACE-inhibitor, Aldosterone Receptor Antagonist, Mineralocorticoid receptor antagonist, beta-blocker. Plasma concentration of Galectin 3 were not associated to albuminuria in linear univariate analyses (Beta: 0.042; 95%CI: −0.018–0.102; *P* = 0.170)

Table 2 illustrates the associations between both Gal-3 and echocardiographic variables and the associations between Gal-3 and cardiovascular biomarkers. In univariate analysis both the echocardiographic variables: TAPSE, E/e’, s’ medial, LAESVindex and the biomarkers: NT-proBNP, proANP, Chromogranin A, Copeptin, hsCRP and Troponin I were associated with Gal-3. In multivariate analysis, adjusted for age, gender, eGFR, treatment with ACE-inhibitor, Aldosterone Receptor Antagonist, Mineralocorticoid receptor antagonist, beta-blocker, NT-proBNP (β: 0.047; 95 % CI: 0.008–0.086; *P* = 0.020), proANP (β: 0.137; 95 % CI: 0.067–0.207; *P* < 0.001), Chromogranin A (β: 0.123; 95 % CI: 0.052–0.194; *P* < 0.001), Copeptin (β: 0.080; 95 % CI 0.000–0.160; *P* = 0.049) and hsCRP (β: 0.051; 95 % CI: 0.001–0.101; *P* = 0.047) were still associated with Gal-3 (Fig. 2). Patients with Gal-3 plasma concentrations above median had markedly lower eGFR (55 vs. 80 ml/min, *P* < 0.001). This association remained significant in multivariate linear regression with Gal-3 as response variable and eGFR as explanatory variable (β: −0.010; 95 % CI −0.012–-0.008; *P* < 0.001), adjusted for age, gender, treatment with ACE-inhibitor, Aldosterone Receptor Antagonist, Mineralocorticoid receptor antagonist, beta-blocker. There were no significant association between Gal-3 and albuminuria (Beat: 0.012; 95 % CI: −0.041–0.065; *P* = 0.652) (Fig. 3).

**Table 2:** Echocardiographic parameters and biomarkers. Linear regression models (response variable: Galectin 3). Multivariate analyses adjusted for age, gender, eGFR, treatment with beta-blocker, ACE-inhibitor, Aldosterone Receptor Antagonist, Mineralocorticoid receptor antagonist, beta-blocker

	Univariate: Beta (95 %-CI)	*P*-value	Multivariate: Beta (95 % CI)	*P*-value
Echocardiographic parameters:				
LVIDd	-0.036 (-0.124–-0.510)	0.410	--	--
LVmassIndex	0.442×10^−3^ (-0.912×10^−3^–1.796×10^−3^)	0.520	--	--
EF biplane	-0.002 (-0.011–0.007)	0.691	--	--
GLS	-0.002 (-0.029–0.024)	0.861	--	--
TAPSE	-0.195 (-0.353–-0.038)	0.015	-0.073 (-0.211–0.065)	0.298
MV decel time	-9.655×10^−5^ (-0.001–0.001)	0.868	--	--
E/e’	0.012 (0.000–0.023)	0.049	0.005 (-0.005–0.014)	0.338
s’ medial	-0.070 (-0.128–-0.011)	0.020	-0.014 (-0.065–0.036)	−0.575
s’ lateral	-0.035 (-0.090–0.019)	0.199	--	--
LAESVi	0.009 (0.001–0.017)	0.021	0.004 (-0.003–0.011)	0.222
Biomarkers:				
Troponin I	0.175 (0.008–0.341)	0.040	0.055 (-0.089–0.198)	0.451
NTproBNP	0.110 (0.072–0.147)	<0.001	0.047 (0.008–0.086)	0.020
proANP	0.320 (0.232–0.407)	<0.001	0.137 (0.067–0.207)	<0.001
ChromagraninA	0.203 (0.125–0.280)	<0.001	0.123 (0.052–0.194)	<0.001
Copeptin	0.211 (0.139–0.282)	<0.001	0.080 (0.000–0.160)	0.049
hsCRP	0.080 (0.021–0.138)	0.008	0.051 (0.001–0.101)	0.047

*Abbreviations*: *LVIDd* Left ventricle internal diameter diastoli, *LVmassIndex* Left ventricle mass index, *EF* Ejection Fraction, *GLS* Global longitudinal strain, *TAPSE* Tricuspid annular plane systolic excursion, *MV decel time* Mitral valve deceleration time, E/e’, *s’* Myocardial peak systolic velocity, *LAESVi* Left atrial end systolic volume index, *NTproBNP* N-terminal pro brain natriuretic peptide, *proANP* Pro atrial natriuretic protein, *hsCRP* High sensitive C-reactive protein, *eGFR* Estimated glomerular filtration rate

## Discussion

The main finding of this present study is threefold. First, plasma concentrations of Gal-3 are associated with neurohumoral activity. Second, plasma concentrations of Gal-3 are associated with renal function evaluated by eGFR, but not with albuminuria. Finally, plasma concentrations of Gal-3 are not associated with myocardial function evaluated by echocardiography in patients with systolic HF.

### Neurohumoral activity and Gal-3

Plasma concentrations of both Gal-3 and NT-proBNP are elevated in HF patients. In the present study we observed significant positive associations between Gal-3 and NT-proBNP and proANP, respectively. These observations are supported by similar findings in other studies [12–14]. Previous studies that also evaluated the long term prognostic value of Gal-3 have demonstrated that the independent prognostic value of Gal-3 disappears after adjusting for NT-proBNP [12, 14, 24–26]. Therefore, although plasma concentrations of Gal-3 are elevated in HF, most likely there are no independently associated with increased mortality risk. This present study is the first to evaluate the relationship of plasma concentrations of Gal-3 and proANP. The positive association is anticipated and confirms the relationship to natriuretic peptides. Natriuretic peptides are part of the neurohumoral activation in HF and markers of increased wall stress in the cardiac cambers. Whether plasma concentrations of Gal-3 are influenced directly by increased wall stress or through neurohumoral activation needs to be examined.

Another cardiac biomarker reflecting neurohumoral activation is Chromogranin A [21, 27]. No prior study has evaluated the relationship between plasma concentrations of Gal-3 and Chromogranin A. Our findings suggest a significantly association between plasma concentrations of Gal-3 and Chromogranin A, thereby reaffirming the relation with the neurohumoral response.

Copeptin, a surrogate marker for vasopressin, involved in volume regulation and electrolyte homeostasis, is also a cardiac biomarker reflecting neurohumoral activity. Copeptin is elevated in HF and predicts mortality risk and risk of re-hospitalization [28, 29]. In the present study plasma concentrations Gal-3 are significantly associated to copeptin. This finding is new and not observed previously.

Another finding of this study is a positive association between plasma concentrations of Gal-3 and elevated plasma concentrations of hsCRP. This finding is in accordance with other studies including HF patients [30, 31]. Gal-3 is also involved in the inflammatory response, and this could explain a positive association between hsCRP and Gal-3.

### Myocardial function and Gal-3

Gal-3 has been linked to cardiac fibrosis in HF, and it would therefore be reasonable to believe that elevated plasma concentrations of Gal-3 would be linked to echocardiographic findings. In the present study of patients with chronic HF and reduced LVEF none of the conventional echocardiographic measures were statistically associated to plasma concentrations of Gal-3. We evaluated both LV systolic (LVEF, global longitudinal strain, s’) and diastolic parameters (E/e’, e’, E/A, LAESVindex), LVmassIndex, LVIDd and right ventricle systolic function (TAPSE).

Few prior studies have systematically evaluated the relationship between echocardiographic measures and plasma concentrations of Gal-3. The DEAL-HF trial performed serial echocardiographic measures in 240 patients with HF reduced LVEF and found a positive association between increased plasma concentrations of Gal-3 and LV remodeling estimated by change in LV end diastolic volume [32]. In a large study of 1440 patients hospitalized with HF plasma concentrations of Gal-3 did not differ significantly between patients with reduced LVEF compared to patients with preserved LVEF after stratifying by New York Heart Association functional class. However, plasma concentrations of Gal-3 were associated LVEF and LV mass index in univariate analysis [14]. Shah et al. [33] examined 115 patients admitted with acute dyspnea, in whom a standard echocardiography had been performed. It was reported that plasma concentrations of Gal-3 were associated to several echocardiographic parameters reflecting higher LV filling pressure and valvular disease (mitral inflow ratio, right ventricular fractional area, right ventricle systolic pressure, more severe mitral-valve or tricuspid-valve regurgitation) [33]. On the contrary other studies have found no association between plasma concentrations of Gal-3 and myocardial function. In a study of 133 chronic HF patients and 45 decompensated HF patients Tang et al. [9] did not observe any association between plasma concentrations of Gal-3 and echocardiographic findings neither systolic (LVEF, LV end systolic diameter, LV end diastolic diameter, LVmassIndex) nor diastolic (mitral inflow ratio, e’, left atrial volume index). Weir et al. [34] examined 100 patients with LV dysfunction after acute myocardial infarction and performed cardiac magnetic resonance imaging at baseline and at 24-week follow-up. Plasma concentrations of Gal-3 were associated to biomarkers reflecting cardiac remodeling, however, there were no association to myocardial function evaluated by cardiac MRI at baseline or change in parameters over time. It is uncertain whether plasma concentrations of Gal-3 reflect myocardial function, and based on previous and the present study plasma concentrations of Gal-3 should likely not play a significantly role in the evaluation of systolic or diastolic function of the LV in HF.

### Renal function and Gal-3

The relationship between plasma concentrations of Gal-3 and eGFR is well established in both HF patients and the general population. Gal-3 has been accepted as a new cardiac biomarker in HF, but also carries potential to be a renal biomarker due to the close association between plasma concentrations of Gal-3 and eGFR.

In the present as in previous studies a negative association between plasma concentrations of Gal-3 and eGFR in HF patients was observed [9, 14]. Moreover, Zamora et al. [24] observed that the prognostic value of plasma concentrations of Gal-3 disappeared after adjusting for renal function. Gopal et al. [35] examined the relationship between plasma concentrations of Gal-3 and renal function in patients with HF and controls. They found plasma concentrations of Gal-3 to be correlated to eGFR in both groups, but Gal-3 were not related to LVEF or clinical congestion.

No previous studies have examined the relationship between albuminuria and plasma concentrations of Gal-3 in HF patients. In patients with impaired renal function O’Seaghdha et al. [10] found no association between albuminuria and plasma concentrations of Gal-3. Meijers et al. [36] have studied the excretion of Gal-3 in both animal models and in HF and dialysis patients. They observed increased plasma concentrations of Gal-3 in HF patients, but they did not observe an increased urinary excretion. They propose that renal clearance of plasma Gal-3 is impaired in HF patients, which explains the elevated plasma concentrations of Gal-3 and the association between renal function and plasma concentrations of Gal-3 [36]. This supports the finding in the present study, where no association was observed between plasma concentrations of Gal-3 and albuminuria. Albuminuria and plasma concentrations of Gal-3 may, therefore, reflect different pathology in the kidneys.

### Methodological considerations and perspectives

Some methodological limitations of this study should be addressed. It is a single center study with a limited sample size. Patients in this study have a short history of HF, they have been in clinically stable through the past 3 months and not all patients have been fully up-titrated in recommended HF treatment. Therefore the results cannot be extrapolated to other groups of patients or other stages of heart failure. This study was conducted as a cross sectional study without follow-up. Therefore, any firm conclusion concerning causal relationship between Gal-3 and NT-proBNP and vice versa has to be interpreted with caution. In this study we measured Troponin I, and no subsequent analyses for high sensitive troponin were made. Due to missing follow-up information we were unable to evaluate the prognostic value of Gal-3.

Plasma concentrations of cardiac biomarkers with renal excretion must be interpreted with caution in patients with renal dysfunction, as increased levels could reflect both decreased clearance and increased cardiac secretion.

While further clinical studies will be necessary to establish the full benefit of Gal-3 as a cardiac biomarker in HF, due to its close relationship with eGFR and NT-proBNP, our studies supports that Gal-3 is elevated in more severe HF and related to other risk markers. The close relationship with eGFR should be evaluated to create a better understanding of this relationship. Future studies could evaluate whether Gal-3 is a cardio-renal marker that e.g. increases before plasma concentrations of creatinine during an acute HF event, a scenario where a new biomarker is needed. And since no clear association has been found between Gal-3 and echocardiographic parameters, future studies should explore the association between plasma concentrations of Gal-3 and myocardial fibrosis e.g. by late gate enhancement with magnetic resonance scanning or by fibrosis identified by myocardial biopsy.

## Conclusion

Increased plasma concentrations of Gal-3 are associated with reduced eGFR and increased neurohumoral activity (NTproBNP, proANP, Copeptin and Chromogranin A), but not with echocardiographic parameters of myocardial function in patients with systolic HF.

## Abbreviations

‘s, Myocardial peak systolic velocity; A, Peak velocity of atrial filling; Albuminuria, Urine albumin excretion, mg/24 h; CKD-EPI, Chronic Kidney Disease Epidemiology Collaboration; E, Mitral flow peak velocity; É, Myocardial peak early velocity; EDTA, Ethylenediamine tetracetic; eGFR, Estimated Glomerular Filtration Rate; Gal-3, Galectin 3; GLS, Global longitudinal strain; HF, Heart failure; hsCRP, High sensitive C-reactive peptide; LAESVindex, Left atrial end systolic index; LV, Left Ventricular; LVEF, Left ventricle Ejection fraction; LVIDd, Left ventricle inter diameter diastolic; NT-proBNP, Aminoterminal pro-brain natriuretic peptide; ProANP, Pro-atrial natriuretic peptide.

## Additional files

Additional file 1: Table S1. Plasma concentration of Galectin-3 according to Age, NYHA class and chronic kidney disease (CKD). (DOCX 13 kb) Additional file 2: A: Comparison of plasma Galectin-3 in patients younger or equal to 70 years vs. older than 70 years, *P* = 0.091. B: Kruskal-Wallis test: *P* = 0.0116. Comparison of plasma Galectin-3 in patients with NYHA I + II vs NYHA III + IV, *P* = 0.234. C: Comparison of plasma Galectin-3 in patients with or without chronic kidney disease (defined as eGFR above or below 60 ml/min/m^2^), *P* < 0.001. (ZIP 42 kb)
