# Supplementary material for: Galectin 3: association to neurohumoral activity, echocardiographic parameters and renal function in outpatients with heart failure
Source: BMC Cardiovasc Disord. 2016 May 31;16:117. doi: 10.1186/s12872-016-0290-7 (PMC4886419; doi:10.1186/s12872-016-0290-7)
Supplement: Additional file 1: Table S1. — Plasma concentration of Galectin-3 according to Age, NYHA class and chronic kidney disease (CKD). (DOCX 13 kb) [file 12872_2016_290_MOESM1_ESM.docx]

**Supplement table.**

**Plasma concentration of Galectin-3 according to Age, NYHA class and chronic kidney disease (CKD).**

**A:**

Comparison of plasma Galectin-3 in patients younger or equal to 70 years vs. older than 70 years, P = 0.091.

**B:**

Kruskal-Wallis test: P=0.0116

Comparison of plasma Galectin-3 in patients with NYHA I+II vs NYHA III+IV, P = 0.234

**C:**

Comparison of plasma Galectin-3 in patients with or without chronic kidney disease (defined as eGFR above or below 60ml/min/m^2^), P < 0.001.
