# Supplementary figures and images for: Galectin 3: association to neurohumoral activity, echocardiographic parameters and renal function in outpatients with heart failure
Source: BMC Cardiovasc Disord. 2016 May 31;16:117. doi: 10.1186/s12872-016-0290-7 (PMC4886419; doi:10.1186/s12872-016-0290-7)

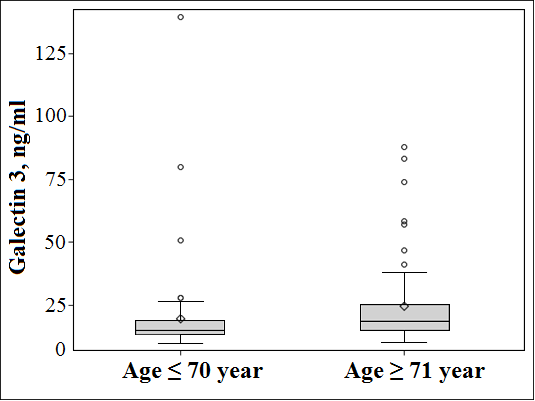

Supplement: Additional file 2: — A: Comparison of plasma Galectin-3 in patients younger or equal to 70 years vs. older than 70 years, P = 0.091. B: Kruskal-Wallis test: P = 0.0116. Comparison of plasma Galectin-3 in patients with NYHA I + II vs NYHA III + IV, P = 0.234. C: Comparison of plasma Galectin-3 in patients with or without chronic kidney disease (defined as eGFR above or below 60 ml/min/m2), P < 0.001. (ZIP 42 kb) [file 12872_2016_290_MOESM2_ESM.zip › Alder_medianR1.png]

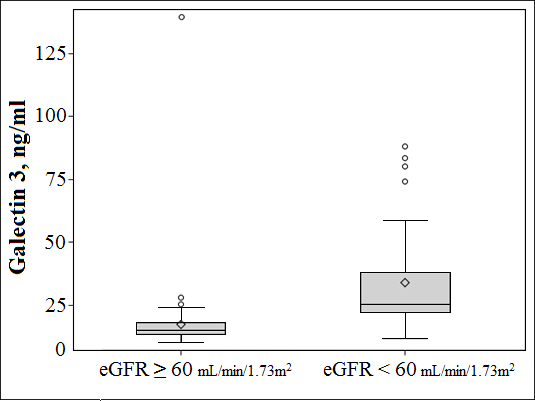

Supplement: Additional file 2: — A: Comparison of plasma Galectin-3 in patients younger or equal to 70 years vs. older than 70 years, P = 0.091. B: Kruskal-Wallis test: P = 0.0116. Comparison of plasma Galectin-3 in patients with NYHA I + II vs NYHA III + IV, P = 0.234. C: Comparison of plasma Galectin-3 in patients with or without chronic kidney disease (defined as eGFR above or below 60 ml/min/m2), P < 0.001. (ZIP 42 kb) [file 12872_2016_290_MOESM2_ESM.zip › CKD_galectin3R1.png]

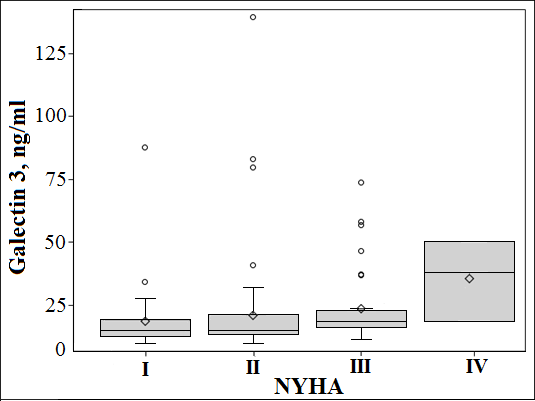

Supplement: Additional file 2: — A: Comparison of plasma Galectin-3 in patients younger or equal to 70 years vs. older than 70 years, P = 0.091. B: Kruskal-Wallis test: P = 0.0116. Comparison of plasma Galectin-3 in patients with NYHA I + II vs NYHA III + IV, P = 0.234. C: Comparison of plasma Galectin-3 in patients with or without chronic kidney disease (defined as eGFR above or below 60 ml/min/m2), P < 0.001. (ZIP 42 kb) [file 12872_2016_290_MOESM2_ESM.zip › NYHA_galectinR1.png]
